# Combined Ultrasound and Fluoroscopy versus Ultrasound versus Fluoroscopy-Guided Caudal Epidural Steroid Injection for the Treatment of Unilateral Lower Lumbar Radicular Pain: A Retrospective Comparative Study

**DOI:** 10.3390/medicina60050809

**Published:** 2024-05-15

**Authors:** Dong yuk Lee, Yongbum Park, Jun Hyeong Song, Jaeki Ahn, Kyung Hwan Cho, Suyeon Kim

**Affiliations:** Department of Rehabilitation Medicine, Sanggye Paik Hospital, Inje University College of Medicine, Seoul 01757, Republic of Korea; s4477@paik.ac.kr (D.y.L.); s1812@paik.ac.kr (J.A.); s4657@paik.ac.kr (K.H.C.); s4766@paik.ac.kr (S.K.)

**Keywords:** ultrasound, fluoroscopy, radiculopathy, caudal, injections, epidural

## Abstract

*Background and Objectives*: This study aimed to evaluate the mid-term effectiveness and safety of a combined ultrasound (US) and fluoroscopy (FL)-guided approach in comparison to US-guided and FL-guided caudal epidural steroid injections (CESI) for treating unilateral lower lumbar radicular pain. *Materials and Methods*: A total of 154 patients who underwent CESI between 2018 and 2022 were included. Patients were categorized into three groups based on the guidance method: combined US and FL (n = 51), US-guided (n = 51), and FL-guided (n = 52). The study design was retrospective case-controlled, utilizing patient charts and standardized forms to assess clinical outcomes, adverse events, complications during the procedures. *Results*: In all groups, Oswestry Disability Index and Verbal Numeric Scale scores improved at 1, 3, and 6 months after the last injection, with no significant differences between groups (*p* < 0.05). The treatment success rate at all time points was also similar among the groups. Logistic regression analysis showed that injection method, cause, sex, age, number of injections, and pain duration did not independently predict treatment success. Blood was aspirated before injection in 2% (n = 1), 13.5% (n = 7), and 4% (n = 2) of patients in the combined US and FL groups, FL-guided groups, and US-guided groups, respectively. Intravascular contrast spread was detected in one patient in the combined method groups and seven in the FL-guided groups. *Conclusions*: When comparing pain reduction and functional improvement, there was no significant difference between the three methods. The combined method took less time compared to using FL alone. The combined approach also showed a lower occurrence of intravascular injection compared to using FL alone. Moreover, blood vessels at the injection site can be identified with an ultrasound using the combined method. Given these advantages, it might be advisable to prioritize the combined US- and FL-guided therapy when administering CESI for patients with unilateral lumbar radicular pain.

## 1. Introduction

Ultrasound (US)-guided caudal epidural steroid injection (CESI) effectively relieves radiating pain from lumbar origin causes [1,2]. US offers advantages such as user-friendliness, adaptability, and the provision of real-time needle guidance without radiation exposure [1,2,3]. Comparative studies have revealed that US-guided CESI demonstrates comparable short-term treatment efficacy, functional outcomes, and patient satisfaction compared to fluoroscopy (FL) guidance [1,2].

Nevertheless, certain precautions must be taken when performing CESI, especially considering the sacral canal diameter and anatomical variations. If the sacral canal’s diameter is less than 2 mm, the use of a needle with a diameter greater than 22 gauge may lead to insertion failure through the sacral hiatus. Additionally, research has indicated that around 1.4% of the human population lacks sacral canal openings or presents with a closed sacral hiatus [4]. In such cases, US assessment of anatomical variations before CESI minimizes procedural failures and enhances patient comfort.

Compared to FL guidance, US guidance does present certain limitations. Notably, it may not detect faulty injections, leading to reduced treatment effectiveness [1,2]. Additionally, the identification of inadvertent intravascular injections is challenging [1,2]. In response to these drawbacks, the combined approach of using both US and FL has been advocated.

Combined FL- and US-guided interventions for musculoskeletal pain treatment have been extensively explored in various studies [5,6,7]. This combined method offers the advantage of avoiding critical structures along the needle path while simultaneously providing real-time visualization of the needle’s progress under US guidance [8]. Furthermore, the use of contrast media during injection allows verification of the accuracy and appropriateness of the injection site through fluoroscopic imaging [8]. Consequently, this combined approach ensures a safer and more precise injection process.

However, there is currently a lack of published literature concerning the combined method specifically tailored to address unilateral lower lumbar radicular pain. Hence, the principal aim of this research was to evaluate the mid-term effectiveness and safety of the combined US and FL method in comparison to both US- and FL-guided CESI, specifically for treating unilateral lower lumbar radicular pain.

## 2. Materials and Methods

### 2.1. Study Design

The current research constitutes a case-controlled, retrospective study, drawing data from patient charts. Throughout the research process, utmost attention was given to preserving patient privacy and ensuring data confidentiality. Ethical clearance for this study was granted by the Institutional Review Board of Inje University Sanggye Paik Hospital (IRB no. 2022-05-014-001), allowing for its implementation. The approval encompassed a Waiver of Informed Consent because the research did not involve direct interaction with the study population, and all patient-identifying details were promptly removed from the dataset upon initial data collection.

### 2.2. Subject

Potential study participants were drawn from patients who had received CESI at our outpatient rehabilitation department between 2018 and 2022. They received either combined US- and FL-guided CESI, US-guided CESI, or FL-guided CESI for unilateral lumbar radicular pain. At the time of the visit, patients made their own choice of guidance method after the medical staff fully explained the pros and cons of each method.

Before the procedure, all patients were requested to complete self-assessment questionnaires, providing baseline information, including pain levels and functional status. Retrospectively, electronic clinical records and questionnaire responses were reviewed to collect data and ensure adherence to the study’s inclusion criteria. To ensure the homogeneity of the study population and minimize potential confounding variables, patients with multi-radicular pain were excluded during the diagnostic process. By implementing these exclusion criteria and focusing solely on patients with unilateral lower lumbar radicular pain, we aimed to provide a clearer understanding of the comparative effectiveness of different CESI guidance methods within this specific patient cohort.

The diagnosis of unilateral lower lumbar radicular pain resulting from spinal canal stenosis or herniated disc was based on comprehensive clinical profiles, physical examinations, electromyography, and imaging studies, including CT scans or MRIs.

Patients meeting the inclusion criteria for this study must have a diagnosis of unilateral lumbar radicular pain attributed to either herniated lumbar disc or spinal stenosis, confirmed through clinical evaluation, electromyography, and imaging studies. Symptoms should persist for a minimum of 3 months, with self-reported back pain and radicular pain scores on the verbal numeric scale (VNS) reaching at least 5 points. Additionally, patients must have failed to achieve sufficient relief after undergoing a minimum of 4 weeks of conservative treatments, such as analgesics and physical therapy. Those who have not previously undergone caudal epidural steroid injection (CESI) or have not received it within a year prior to the study period are eligible. Complete medical records and follow-up data must be available for inclusion.

Patients meeting any of the exclusion criteria for this study were excluded from participating. These criteria included incomplete medical records or missing follow-up data, a history of substance abuse or dependence, psychiatric disorders, or cognitive impairment that may impact accurate self-reporting of pain and functional outcomes. Additionally, patients with a history of previous lumbar spine surgeries and underlying concurrent medical conditions, such as uncontrolled diabetes or autoimmune disorders, which could affect treatment outcomes or confound the efficacy of caudal epidural steroid injection (CESI), were excluded. Patients with contraindications to CESI, such as active infection at the injection site, coagulopathy, or allergy to injectable medications, were also excluded, along with pregnant patients or those planning pregnancy during the study period, individuals under 18 years of age, and those presenting with progressive motor deficits or significant sensory deficits.

### 2.3. Combined US- and FL-Guided CESI

Interventions were performed by experienced physicians, Park Y and Ahn JK, each with over 15 years of expertise in US- and FL-guided interventions. All treatments were carried out on an outpatient basis. This study was conducted with reference to the US- and FL-guided CESI procedure of K. D Park et al. [1] and Y. Park et al. [2]. The procedure description in this paper partly reproduces their wording.

**Figure 1:**
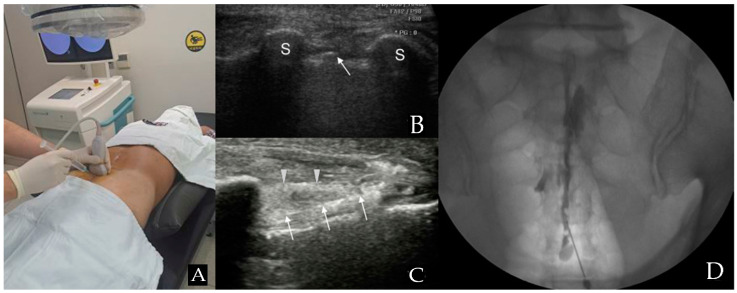
Combined ultrasound and fluoroscopy-guided caudal epidural steroid injection. (**A**) Utilizing ultrasound transverse view to insert the spinal needle in the appropriate location. (**B**) Ultrasound image seen from the probe in (**A**) position. The arrow points to the caudal epidural needle, which is located between the two sacral cornua, indicated by S (hyperechoic reversed U shape) in the image. (**C**) A view along the longitudinal axis displays the needle within the caudal epidural space. The arrowhead is pointing to the sacrococcygeal ligament, while the arrow points to the caudal epidural needle. (**D**) An anteroposterior X-ray reveals the contrast medium’s epidural spread following the administration of a caudal epidural injection.

Patients were positioned prone, and the RS80A (Samsung Medison Co., Ltd., Seoul, Republic of Korea) with a linear array transducer (3–12 MHz) was used for US imaging. Before injection, US was used to verify anatomical variations, ensuring proper needle length and diameter selection for the caudal portal. The US transducer was initially positioned at the midline to acquire a transverse view of the sacral hiatus [9,10] (Figure 1A). The sacral hiatus was identified in the US images based on specific landmarks. In the transverse section, the two sacral cornua appeared as two hyperechoic structures, forming an inverted U shape [2,9,10,11]. Additionally, color Doppler imaging was used to identify blood vessels.

With the assistance of another medical staff member, the interventionalist, wearing sterile gloves, arranged the equipment on a sterile covering. The sacrococcygeal area was sterilized with iodine-based povidone and alcohol solutions. The location of the sacral cornu was confirmed through palpation. Finally, the needle was inserted toward the affected side to deliver the medication toward the specific side and optimize the medication’s chances of reaching the anomaly site [2]. A 22-gauge, 90 mm spinal needle (SP.QB, Taechang Industrial Co., Ltd., Chungnam, Republic of Korea) was then inserted between the two cornua into the sacral hiatus (Figure 1B). Once the sacrococcygeal ligament was successfully penetrated, often accompanied by a pop or give sensation, the transducer was rotated to attain a longitudinal view of the sacrum and sacral hiatus. The needle was then advanced into the sacral canal under real-time US guidance (Figure 1C) [2,9,10,11].

After needle insertion, it was crucial to verify the absence of blood in the syringe before proceeding. If blood was present or there were suspicions of cerebrospinal fluid (CSF), an inhalation test was conducted. If either blood or CSF was present, the needle was cautiously adjusted.

To ensure precise needle placement and epidural flow and to avoid intravascular, intradural, or soft tissue infiltration, approximately 1 mL of contrast media (GE Healthcare Ireland Limited, GE Healthcare (Shanghai, China) Co., Ltd.) was injected before administering the medication (Figure 1D). After a careful review of the epidurogram, a test dose of 1–2 mL of 1% lidocaine was injected. After injecting a test dose, patients were closely observed for immediate side effects, including metallic taste, drowsiness, confusion, tachycardia, hypotension, nausea, tinnitus, numbness, indistinct speech, and gait disturbance, for 1 to 2 min [12]. After confirming the absence of any abnormal findings, the injection consisting of 20 mL (0.5% lidocaine 18.0 mL + dexamethasone 10 mg 2 mL) was injected.

### 2.4. US-Guided CESI

Ultrasound was utilized following the same sequence as previously described for the combined US- and FL-guided CESI. Without using FL, the interventionalist inserted a 22-gauge, 90 mm spinal needle (SP.QB, Taechang Industrial Co., Ltd., Chungnam, Republic of Korea) toward the side of pain to precisely deliver medication to the lesion site, optimizing effectiveness [13]. An inhalation test was performed in the same way as the combined method. Following verification of blood and CSF absence, a test dose of 1–2 mL of 1% lidocaine was administered. During the lidocaine test, color Doppler was employed to check whether or not intravascular spread was present. When unidirectional flow expressed in one distinct color was observed, it was considered a positive spectrum. Flow in other directions, represented by various colors, should not be observed [11]. If flow in other directions was observed, the needle was readjusted using US guidance, confirmed by Doppler spectrum change. Finally, the injection was performed using the same regimen as the combined method.

### 2.5. FL-Guided CESI

The patients were positioned in a prone manner on a fluoroscopic table. To enhance visibility of the sacral hiatus, a pillow was placed under the hip to tilt the pelvis. Preparation of the sacrococcygeal area involved the application of an iodine-based povidone solution and an alcohol solution. Confirmation of the location of the sacral cornu was performed in the same manner as the previous method. The anteroposterior and lateral view was adjusted for better visualization of the sacral hiatus. Utilizing FL guidance, the same needle used in the combined method was carefully inserted into the epidural space.

Then, an inhalation test was performed in the same way as the previous method. To ensure precise needle placement and epidural flow, 1 mL of contrast media (GE Healthcare (Shanghai, China) Co.) was given. After thoroughly reviewing the patient’s epidurogram, the lidocaine test dose injection and injection of the same regimen were performed in the same manner as the previous method.

Patients in all groups visited the hospital 2 weeks after the initial injection for reevaluation, and a decision was made on whether to receive additional injections. In cases where the patient’s pain relief is less than a 50% reduction in VNS, a second injection was scheduled. However, if the initial injection led to a substantial alleviation of symptoms (VNS reduction ≥ 50%), the second injection was omitted. If there was no observed pain relief or a deterioration of pain, a second injection was not pursued. The decision about whether or not to perform additional injections followed the research method of K. D Park et al. [1], Y. Park et al. [2], and J. H. Jang et al. [14]. All patients had previously received at least a 4-week course of conservative treatments without any significant improvement. Therefore, there were no imposed restrictions on the persistence of prior exercise routines, medication, or work-related activities. Other than general conservative treatment, no other invasive treatment was performed.

### 2.6. Review of the Clinical Data

To extract comprehensive data regarding demographics, therapeutic interventions, pain severity, utilization of pain relief medications, and evaluations of functionality, a standardized chart abstraction form was employed. Clinical effectiveness was evaluated using the VNS and the Oswestry Disability Index (ODI) at various time points: pretreatment and at 1, 3, and 6 months post-final injection. The VNS is a pain assessment scale ranging from 0 (pain free) to 10 (worst imaginable pain) [15]. The ODI is a widely utilized disease-specific measure for patients with lower back pain [16]. It comprises ten items; each score ranges from 0 to 5, and the cumulative score is subsequently doubled, resulting in an ODI range from 0 to 100. Independent variables were recorded, including injection technique, the quantity of injections administered, the underlying cause of radicular pain, pain duration, sex, and age. Age was categorized into five groups: younger than 39 years old, 40–49, 50–59, 60–69, and older than 70 [1]. Radiating pain duration was divided as acute and subacute (under 6 months) or chronic (over 6 months) [17].

Effective treatment was defined as a reduction of 2.5 points or more in the VNS score and a decrease of 10 points or greater in the ODI score at 1, 3, and 6 months [18]. Patients who did not meet the specified criteria or required additional CESI or surgical treatment during the follow-up interval were considered non-responders. The data for VNS and ODI scores from these non-responders were confirmed for statistical analysis but excluded thereafter. Patients who achieved successful outcomes, meeting the defined criteria, were identified as having undergone successful treatment and were qualified for further follow-up. During the study, we meticulously reviewed the medical charts for immediate adverse events, such as temporary pain or discomfort at the injection site, temporary numbness or weakness in the legs, flushing, or warmth [1]. Patients were provided with a questionnaire after the procedure to assess potential complications, to be completed within 48 h after the injection and returned at the next visit (14 days after first injection). The questionnaire checked severe complications, including headache, fever, pain aggravation, hematoma, and infection, allowing for effective monitoring and management of adverse events.

### 2.7. Statistics

Pearson’s chi-square test and one-way analysis of variance (ANOVA) were employed for comparing variables such as sex, age, body mass index (BMI), number of injections, analgesic use, anticoagulant use, and pain duration among the three groups. This research employs statistics and compares the variables used by J. H. Jang et al. [14]. The description of the statistics partially replicates their wording. At every point in the evaluation of VNS and ODI scores, scores were compared through repeated measures ANOVA, with post hoc comparisons conducted using Bonferroni’s correction. Pearson’s chi-square test was applied for examining disparities in proportions, while one-way ANOVA was used to compare the procedure duration among groups. Univariate and multivariate logistic regression analyses, alongside Pearson’s chi-square test, were employed to assess whether factors such as the injection technique, age, sex, analgesic usage, and the quantity of injections served as independent predictors of treatment effectiveness. All statistical assessments were conducted using SAS Enterprise Guide 4.1 (version 4.1.0.471). A *p*-value below 0.05 was regarded as statistically significant.

## 3. Results

**Figure 2:**
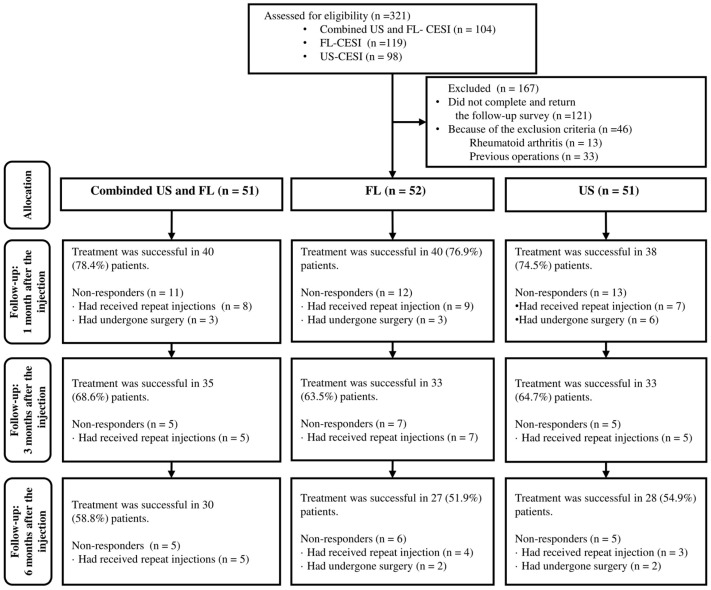
Flow chart of the patients.

A total of 321 patients received CESI, with 104, 98, and 119 receiving combined US- and FL-guided or US- or FL-guided CESI, respectively. Among these, 121 (37.7%) patients were ineligible due to incomplete or unsubmitted follow-up surveys, and 46 (14.3%) were excluded based on the exclusion criteria. Additionally, 33 patients (10.2%) with prior surgeries and 13 (4.0%) with certain underlying diseases were excluded. Eventually, 154 (48%) patients, including 51, 52, and 51 who received combined US and FL, FL-guided and US-guided CESI, respectively, were included (Figure 2).

The average age of patients in the combined US and FL, FL-guided and US-guided CESI was 52.84 ± 12.00, 55.73 ± 10.21, and 56.57 ± 9.30 years old, respectively, with no significant differences among the three groups. Moreover, there were no significant intergroup differences in sex, BMI, the number of injections, the etiology (herniated lumbar disc or stenosis), intended site of action (nerve root), analgesic use, and pain duration (Table 1).

**Figure 3:**
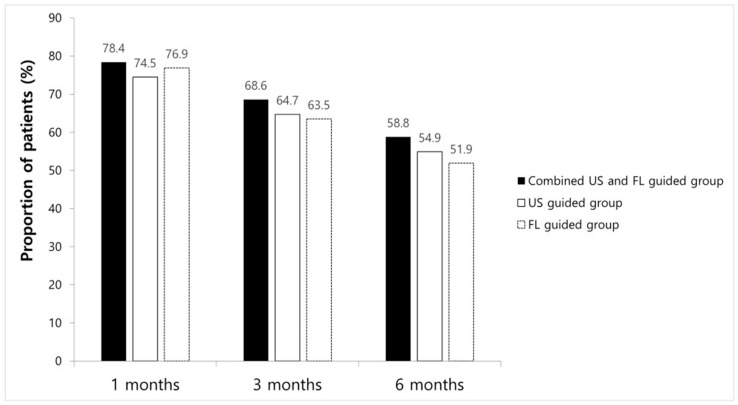
Chart showing success rate at 1 month, 3 months, and 6 months in each group.

ODI and VNS scores at all time points improved significantly compared to the baseline in every group. However, no significant differences between groups were found (Table 2). The proportions of patients with a VNS score improvement of ≥2.5 points and an NDI score improvement of ≥10 points are shown in Figure 3; there were no significant intergroup differences at any time point. After 1 month, eight patients received additional injections, while three had surgical interventions in combined method groups, resulting in a 1-month treatment success rate of 78.4% (n = 40). In the FL-guided and US-guided groups, nine and seven patients, respectively, had received repeat injections, and three and six patients, respectively, had undergone surgery at 1 month. Thus, the 1-month treatment success rates were 76.9% (n = 40) and 74.5% (n = 38), respectively. By the 3-month mark, five patients in the combined US and FL group, seven in the FL-guided group, and five in the US-guided group had received additional interventions, with success rates of 68.6% (n = 35), 63.5% (n = 33), and 64.7% (n = 33), respectively. At the 6-month follow-up, five patients in the combined US and FL group received additional injections, while in the FL-guided group, four received extra injections, and two underwent surgery. Similarly, in the US-guided group, three patients received additional injections, and two opted for surgery. Therefore, the final 6-month treatment success rates were 58.8% (n = 30), 51.9% (n = 27), and 54.9% (n = 28), respectively (Figure 3). There were no significant differences in the success rate at any given time point among the three groups.

Univariate and multivariate logistic regression analyses demonstrated that the injection technique, the etiology (herniated lumbar disc, stenosis), pain duration, sex, age, the number of injections, and analgesic usage were not independent predictors of treatment success (*p* > 0.05; Table 3 and Table 4). Moreover, there was no clinically significant difference between the three groups in the proportion of analgesic (NSAIDs and opioid) users during the 6-month follow-up period. The procedure time of the combined US and FL (274 s) and US-guided method (247 s) was less than the FL-guided method (389 s).

Immediately after the procedure, four patients in the combined US and FL group, three in the FL-guided group, and two in the US-guided group experienced vasovagal reactions, while four, three, and five patients, respectively, developed a transient headache. During the 14-day observation period after injection, one patient in the combined US and FL group, three in the FL-guided group, and three in the US-guided group complained of a brief worsening of pain (lasting less than 48 h) after the procedure. None of the patients reported headaches indicating post-dural puncture syndrome, and there were no instances of infection or hematoma. Blood was aspirated before injection in 2% (n = 1), 13.5% (n = 7), and 4% (n = 2) of patients in the combined US and FL groups, FL-guided groups, and US-guided groups, respectively. Intravascular contrast spread was noted in one patient in the combined US and FL group and seven in the FL-guided groups.

## 4. Discussion

In this retrospective investigation, we included 154 patients who underwent CESI, utilizing different guidance methods, for the management of unilateral lumbar radicular pain. We examined the outcomes of three distinct techniques: combined US and FL-guided CESI, US-guided CESI, and FL-guided CESI. Treatment success rates were 58.8% (n = 30), 54.9% (n = 28) and 51.9% (n = 27), respectively. All three guidance methods were effective in reducing pain and improving patient functionality, with no significant differences observed among the groups.

Our findings align with the existing literature, indicating the effectiveness of both US- and FL-guided CESI for managing lumbar radicular pain [1,2]. The combined US- and FL-guided CESI method, while less common in the literature, demonstrated similar effectiveness in our study. This suggests that the combined approach can offer comparable outcomes to traditional guidance methods.

This study pioneers the evaluation and comparison of treatment efficacy and injection efficiency among combined US- and FL-guided, FL-guided, and US-guided CESI throughout a 6-month monitoring period among patients with unilateral lower lumbar radicular pain. Factors such as procedure time, adverse events, and complications were also considered. In terms of procedure time, combined US- and FL-guided CESI and US-guided CESI had shorter durations compared to FL-guided CESI. This observation aligns with the established notion that US guidance can expedite the procedure, possibly increasing its efficiency and patient comfort [9]. Additionally, the adverse events and complications observed in the three groups were generally manageable and infrequent, with no remarkable differences. Furthermore, the combined US and FL approach demonstrated a reduced incidence of intravascular injection compared to the FL-guided method.

CESI has firmly established its role in both the treatment and examination of patients with lumbar radicular pain, particularly in cases refractory to analgesia and physiotherapy [19]. CESI offers a convenient and safe means of drug administration within an outpatient setting, presenting a diminished risk of thecal sac puncture and inadvertent intrathecal injection [20]. Despite the proficiency of practitioners, incorrect needle placement has been documented in a considerable 25% to 36% of cases during unassisted procedures [21]. Therefore, FL-guided CESI has emerged as valuable for confirming precise caudal epidural needle placement [22,23]. This method enables visualization of the target region and contrast spread. However, despite its popularity, the employment of FL raises concerns primarily due to radiation exposure.

Recent studies have explored the utility of US in the context of CESI [1,2,3]. US presents several advantages, including its user-friendliness, radiation-free nature, and adaptability in various clinical environments [24]. A significant merit of US lies in its capacity to deliver real-time, continuous needle guidance images [3]. Klocke et al. [9] first reported the incorporation of US in CESI, emphasizing its specific benefits in moderately obese patients and individuals with difficulty in adopting a prone position. Further reinforcing the efficacy of US, Chen et al. [3] conducted an evaluation of US-guided CESI in a cohort of 70 patients experiencing radicular pain, achieving a 100% success rate in needle placement. The study utilized a high-frequency transducer to accurately identify the sacral hiatus during injection.

Beyond accuracy and feasibility, US offers the added advantage of producing clear images of the sacral hiatus and its anatomical variations, which are often responsible for complicating or even precluding CESI [25]. In previous investigations, approximately 1–2% of the examined population exhibited closed sacral canals, rendering CESI unfeasible [4,21]. These anatomical variations are not discernible through the use of FL. The integration of US addresses this limitation, enabling a more comprehensive understanding of anatomical diversity and thereby enhancing the potential success of CESI procedures.

Apart from anatomical variations, the measurement of the sacral canal diameter at the apex of the sacral hiatus should also be considered. Clarista et al. [26] revealed that the sacral canal diameter was less than 2 mm in 1% of cases, while another study indicated such dimensions in 6.25% of cases [25]. Instances where the diameter falls below 2 mm are associated with notable patient discomfort, including severe soreness and pain at the injection site. This discomfort arises when the injection needle barely breaches the sacrococcygeal ligament, accompanied by pronounced resistance during the injection procedure, making medication delivery almost impractical [21].

It is important to recognize that a needle diameter surpassing 22 gauge could lead to insertion failure through the sacral hiatus, especially when the hiatus measures less than 2 mm. Chen et al. [21] reported four patients with sacral canal diameters ranging from 1.2 mm to 1.6 mm, experiencing failed injections. Similarly, Park et al. [2] reported injection failures in two patients who underwent FL-guided CESI. Ultrasound measurements of the sacral hiatus for these patients showed diameters less than 2 mm, specifically 1.32 mm and 1.27 mm [2]. Consequently, the acquisition of anatomical information before intervention can mitigate patient discomfort and contribute to a shorter procedure time [2].

The US-guided approach also has drawbacks. One concern is the potential for the drug to be injected in the wrong direction, unlike FL-guided procedures that allow needle adjustment. To address this, experts recommend inserting the needle toward the affected side, aiming to enhance targeted drug delivery [13,27]. However, Lee et al. [28] found that, despite adhering to this recommendation, 13.6% of patients showed a higher drug concentration on the opposite side. Another limitation is the inability to detect intravascular injections, documented at varying rates from 2.5% to 9% [29,30]. While color Doppler and lidocaine test dose injections are employed as safeguards, the US-guided method remains incapable of detecting every intravascular injection [2]. In contrast, the FL-guided approach uses a contrast agent for real-time visualization, solving this limitation.

Numerous studies have explored the efficacy of a combined FL- and US-guided approach in managing musculoskeletal pain, capitalizing on the respective strengths of both methodologies [5,6,7]. A key advantage is its ability to avoid critical structures within the needle path while concurrently monitoring needle advancement in real time under US guidance. Moreover, the injection of contrast media enables confirmation of accurate injection site placement through fluoroscopic imagery. Importantly, the combined FL and US-guided approach could potentially entail a lower dose of radiation exposure compared to using FL alone. While promising, within the domain of treating patients afflicted with unilateral radicular pain, the application of this method remains relatively unexplored. The present study stands as a pioneering effort in this regard, shedding light on the potential of the combined approach for this specific condition.

Considering how new and unique this study is, and because there is not a lot of previous research on this topic, it is clear that more studies are needed to understand the combined FL- and US-guided approach better. This study can inspire and encourage future research that will help improve and broaden our knowledge of how well and how broadly this method works for managing musculoskeletal pain.

This study has a few limitations. Firstly, its retrospective design relies on data collected from past medical records, potentially introducing issues with data accuracy, completeness, and selection bias. Retrospective studies may not provide the same level of control as prospective studies, and the results may be subject to confounding variables not considered in the analysis. Secondly, the sample size in this study is relatively small, especially after exclusions. A larger sample size would enhance the statistical power of the study and improve the generalizability of the results. The sample size might limit the ability to detect subtle differences between the groups if they exist. Third, the paper does not thoroughly account for potential confounding variables, such as whether patients received additional conservative treatment during the follow-up period after treatment, which could affect outcomes. We thought that medication or physical therapy probably did not have much impact because we included patients who had not become better with these treatments. Last, the study only evaluates outcomes up to 6 months after the last injection. Chronic conditions like lumbar radicular pain may require longer follow-up periods to assess the durability of treatment effects and the potential for relapse.

## 5. Conclusions

Our study adds to the discussion on guidance methods for CESI. We found no significant difference in outcomes among the three techniques, suggesting flexibility based on patient needs and resources. The combined method offers real-time imaging for needle guidance and sacral anatomy evaluation using ultrasound. It also utilizes fluoroscopy for accurate needle placement and intravascular injection detection, overcoming ultrasound limitations. Additionally, it reduces procedure time compared to other methods. Therefore, it emerges as a promising option for managing radicular pain, but larger studies with longer follow-ups are needed to validate its long-term efficacy and safety.

## Figures and Tables

**Table 1:** General characteristics of the patients.

	Combined US and FL (n = 51)	FL (n = 52)	US (n = 51)
Age (years)	52.84 ± 12.00	55.73 ± 10.21	56.57 ± 9.30
Sex, n (%)			
Female	37 (72.5)	32 (61.5)	37 (72.5)
Male	14 (27.5)	20 (38.5)	14 (27.5)
BMI (kg/m^2^)	24.09 ± 2.33	24.36 ± 2.99	23.83 ± 2.60
Number of injections	1.43 ± 0.50	1.46 ± 0.50	1.41 ± 0.49
Cause, n (%)			
HLD	18 (35.3)	18 (34.6)	20 (39.2)
Stenosis	33 (64.7)	34 (65.4)	31 (60.8)
Intended site of action (nerve root), n (%)			
L4	27 (52.9)	27 (51.9)	24 (47.1)
L5	17 (33.3)	19 (36.5)	20 (39.2)
S1	7 (13.7)	6 (11.5)	7 (13.7)
Analgesic use, n (%)			
NSAID usage	33 (64.7)	25 (31.1)	20 (54.1)
Opioid usage,	27 (61.4)	21 (51.2)	20 (54.1)
Pain duration (Month)	6.80 ± 2.16	6.61 ± 2.21	6.68 ± 2.05

Values are mean ± standard deviation. US: ultrasound, FL: fluoroscopy, BMI: body mass index, HLD: herniated lumbar disc, NSAIDs: non-steroidal anti-inflammatory drugs.

**Table 2:** Comparison of VNS and NDI scores from the initial assessment to those at 1, 3, and 6 months following the last injection.

		Baseline	1 Month	3 Month	6 Month
	Combined	6.32 ± 1.03	2.65 ± 1.58 *	2.33 ± 1.41 *	2.64± 1.35 *
VNS	US	6.23 ± 0.86	2.75 ± 1.86 *	2.51 ± 1.4 *	2.68 ± 1.95 *
	FL	6.35 ± 1.04	2.88 ± 1.85 *	2.33 ± 1.52 *	2.76 ± 1.52 *
	Combined	24.37 ± 5.30	12.10 ± 5.44 *	11.20 ± 4.18 *	12.97 ± 5.44 *
ODI	US	24.13 ± 5.02	12.65 ± 6.20 *	11.79 ± 5.40 *	13.58 ± 6.3 *
	FL	25.73 ± 5.92	13.94 ± 6.46 *	12.13 ± 5.88 *	13.00 ± 5.56 *

Values are mean ± standard deviation * *p* < 0.05: Comparison prior to and following the injection. VNS: Verbal Numeric Scale, ODI: Oswestry Disability Index, US: ultrasound, FL: fluoroscopy.

**Table 3:** Univariable analysis to explore potential predictors of injection efficacy during the follow-up period.

Characteristic	Responders (n = 85)	Not Responders (n = 69)	*p*-Value
Injection method, n (%)			0.779
Combined	30 (35.3)	21 (30.4)	
US	27 (31.8)	25 (36.2)	
FL	28 (32.9)	23 (33.3)	
Cause, n (%)			0.298
HLD	34 (40.0)	22 (31.9)	
Stenosis	51 (60.0)	47 (68.1)	
Pain duration, n (%)			0.169
<6 month	34 (37.5)	15 (25.9)	
>6 month	27 (62.5)	43 (74.1)	
Sex, n (%)			0.833
Female	43 (67.2)	40 (69.0)	
Male	21 (32.8)	18 (31.0)	
Age, n (%)			0.815
≤39	8 (12.5)	6 (10.3)	
40–49	14 (21.9)	14 (24.1)	
50–59	20 (31.3)	18 (31.0)	
60–69	14 (21.9)	16 (27.6)	
>70	8 (12.5)	4 (6.9)	
Number of injections, n (%)			0.943
1	36 (56.3)	33 (56.9)	
2	28 (43.8)	25 (43.1)	
Analgesic use, n (%)			
NSAID usage	32 (55.2)	38 (59.4)	0.639
Opioid usage	35 (54.7)	33 (56.9)	0.806

US: ultrasound, FL: fluoroscopy, HLD: herniated lumbar disc, NSAIDs: non-steroidal anti-inflammatory drugs.

**Table 4:** Multiple logistic regression analysis to explore potential predictors of injection efficacy during the follow-up period.

Factor	OR	95% CI	*p* Value
US vs. FL -guided methods	0.912	0.582–1.428	0.686
Cause (HLD, stenosis)	0.988	0.463–2.111	0.976
Sex	1.140	0.517–2.513	0.746
Age	1.006	0.972–1.041	0.729
Number of injections	1.062	0.503–2.242	0.875
Pain duration	0.863	0.725–1.028	0.099

US: ultrasound, FL: fluoroscopy, HLD: herniated lumbar disc.

## Data Availability

The data utilized to support the findings of this research can be obtained from the corresponding author upon inquiry.
